# Silver Nanoparticle Modified Electrode Covered by Graphene Oxide for the Enhanced Electrochemical Detection of Dopamine

**DOI:** 10.3390/s17122771

**Published:** 2017-11-29

**Authors:** Jae-Wook Shin, Kyeong-Jun Kim, Jinho Yoon, Jinhee Jo, Waleed Ahmed El-Said, Jeong-Woo Choi

**Affiliations:** 1 Department of Chemical and Biomolecular Engineering, Sogang University, 35 Baekbeom-ro, Mapo-gu, Seoul 04107, Korea; livsuwon@gmail.com (J.-W.S.); sn0wman@naver.com (K.-J.K.); iverson0607@naver.com (J.Y.); jinhee2161@gmail.com (J.J.); waleed@aun.edu.eg (W.A.E.-S.); 2 Chemistry Department, Faculty of Science, Assiut University, Assiut 71516, Egypt; 3 Department of Biomedical Engineering, Sogang University, 35 Baekbeom-ro, Mapo-gu, Seoul 04107, Korea

**Keywords:** silver nanoparticle, neurotransmitter, dopamine, graphene oxide, electrochemical signal

## Abstract

Several neurological disorders such as Alzheimer’s disease and Parkinson’s disease have become a serious impediment to aging people nowadays. One of the efficient methods used to monitor these neurological disorders is the detection of neurotransmitters such as dopamine. Metal materials, such as gold and platinum, are widely used in this electrochemical detection method; however, low sensitivity and linearity at low dopamine concentrations limit the use of these materials. To overcome these limitations, a silver nanoparticle (SNP) modified electrode covered by graphene oxide for the detection of dopamine was newly developed in this study. For the first time, the surface of an indium tin oxide (ITO) electrode was modified using SNPs and graphene oxide sequentially through the electrochemical deposition method. The developed biosensor provided electrochemical signal enhancement at low dopamine concentrations in comparison with previous biosensors. Therefore, our newly developed SNP modified electrode covered by graphene oxide can be used to monitor neurological diseases through electrochemical signal enhancement at low dopamine concentrations.

## 1. Introduction

Dopamine is a key molecule in neurotransmissions in the central and peripheral nervous systems. Therefore, dopamine is responsible for several physiological activities such as behavior, memory and movement [1,2]. An abnormal level of dopamine causes severe neurological diseases, including Parkinson’s disease (PD), schizophrenia and attention deficit hyperactivity disorder (ADHD) [3]. Owing to this critical role of dopamine in the nervous system, numerous studies and technologies have been developed to monitor the level of dopamine in a sensitive and selective manner, which is highly important for early diagnosis of these neurological diseases. High performance liquid chromatography (HPLC) [4], immunoassay [5], spectrophotometric [6] and coulometric [7] methods have been reported to be suitable for dopamine detection. However, most of these techniques require complex steps and procedures, which are expensive, laborious and time-consuming.

To this end, electrochemical dopamine detection techniques have been intensively studied, owing mostly to their simplicity, rapid response time and excellent sensitivity [8,9]. Dopamine is known to be oxidized or reduced at specific electrical potentials and thus, can be effectively measured by electrochemical methods without using any additional redox couples or enzymes. Nevertheless, due to the concentrations of various other neurotransmitters in the body that exist within the range of a picomolar to nanomolar scale, there is a need to increase the sensitivity of electrodes in order to detect dopamine accurately [10]. Traditionally, metal materials like gold and platinum have been widely applied in biosensing applications largely due to their excellence in improving the electrocatalytic property of the electrode [11,12]. However, despite these advantages, gold and platinum are not preferred for use in actual biosensors owing to limitations such as low sensitivity, linearity at a low dopamine concentration and limited resources [13,14].

**Figure 1:**
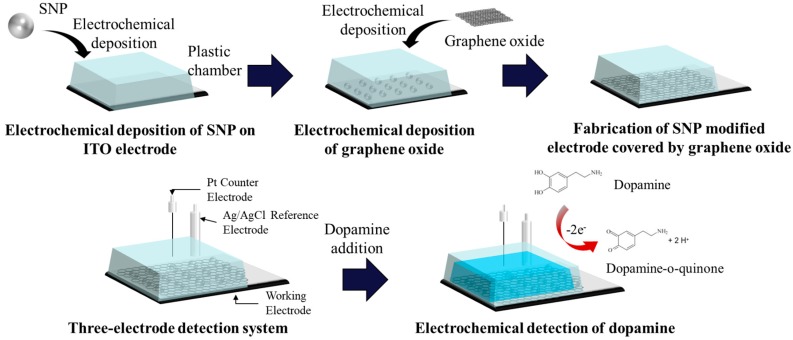
Schematic diagram of an SNP modified electrode covered by graphene oxide and the process of dopamine detection.

Herein, we report upon an electrochemical dopamine biosensor wherein graphene oxide-covered silver nanoparticles (SNPs) were utilized as a core material on an indium tin oxide (ITO) coated electrode (Figure 1). Moreover, for the first time, electrochemically deposited SNPs were covered by a graphene oxide sheet, in contrast to other studies where SNPs were either mixed with or stacked on top of graphene oxide. SNPs are a great alternative metal material due to their low expense and effective electrochemical performance compared to gold and platinum at low dopamine concentrations. Additionally, graphene oxide is another beneficial alternative material choice owing to its almost unlimited availability, good electrocatalytic property and stability. These materials, however, have disadvantages when used separately because of the electrochemical instability of SNPs and the electrical conductivity loss of graphene oxide following a chemical process, which is necessary for the modification of an electrode. Hence, we hypothesized that SNP modification on a conducting working electrode surface in combination with a graphene oxide modification will enhance the electrocatalytic performance and stability of the dopamine biosensor, while the weaknesses of both SNPs and graphene oxide will be hindered. To confirm the improved capability of the SNP modified electrode covered by graphene oxide, we conducted experiments comparing the electrochemical signals among: (1) SNP modified electrodes; (2) ITO electrodes covered by graphene oxide and (3) SNP modified electrodes covered by graphene oxide, using cyclic voltammetry (CV), differential pulse voltammetry (DPV) and the amperometric i-t method.

## 2. Materials and Methods 

### 2.1. Materials

A silver (I) nitrate particle sample was purchased from Daejung Chemical & Metals (Shiheung-City, Korea). Sodium sulfate powder was purchased from Junsei Chemical (Tokyo, Japan). Phosphate buffered saline (PBS) (pH 7.4, 10 mM) solution used as the electrolyte in this study and triton X-100 solution were obtained from Sigma-Aldrich (St. Louis, MO, USA). Single layer graphene oxide (500 mg/L) dispersed in water was purchased from Graphene Supermarket (Calverton, NY, USA). Sylgard (Dow Corning, Midland, MI, USA) 184 silicone elastomer curing agent and Sylgard 184 silicone elastomer base were acquired from Dow Corning (Midland, MI, USA) for polydimethylsiloxane (PDMS) preparation. All aqueous solutions were prepared using deionized water (DIW) from a Millipore Milli-Q water purifier operating at a resistance of 18 MΩ/cm.

### 2.2. Preparation of ITO Electrodes

ITO electrodes (30 mm length × 10 mm width) were cleaned by sonication for 30 min using a triton-X 0.1% solution, DIW and ethanol, sequentially. The washed ITO electrode was then fully dried by N_2_ gas. PDMS, an adhesive material, was prepared by mixing a Sylgard^®^ 184 silicone elastomer base and a Sylgard^®^ 184 silicone elastomer curing agent at a 10:1 ratio. The fully mixed PDMS was placed in a vacuum chamber to remove bubbles and stored at −4 °C to avoid curing. After PDMS preparation, the bottom of a plastic chamber (20 mm length × 10 mm width × 5 mm height) was dipped into the prepared PDMS and excess PDMS was removed to procure the area of attachment of the plastic chamber onto ITO electrode. This plastic chamber was then attached to the ITO electrode. Following this, the plastic chamber attached the ITO electrode was heated in a 70 °C oven for 30 min to fabricate an electrode chamber with cured PDMS.

### 2.3. Electrochemical Deposition of SNPs and Graphene Oxide

The purchased silver (I) nitrate was dissolved in DIW to prepare a 10 mM silver nitrate solution. In order to deposit the SNPs onto the ITO electrode electrochemically, the prepared electrode with the attached chamber was filled with 1 mL of 50 mM silver nitrate solution. The silver nitrate solution in the chamber was well pipetted before the voltage was applied. For the electrochemical deposition of the SNPs onto the electrode, a voltage of −1.3 V was applied to the electrode for 5 s. The graphene oxide solution was diluted to 50 mg/mL using DIW before deposition. This prepared graphene oxide solution was sonicated for 30 min in order to be fully dispersed and centrifuged at 13,000 rpm for 15 min. After centrifugation, sodium sulfate was added as an electrolyte with a 0.1 M concentration. Then, 1 mL of the supernatant of the sonicated and centrifuged graphene oxide was added to the chamber of the SNP-deposited electrode. After that, a voltage of −1.6 V was applied to the electrode for 30 s to obtain the electrochemical deposition of graphene oxide on the electrode. The same method of deposition was used for the fabrication of the ITO electrode covered by graphene oxide. After each electrochemical deposition step, washing of the surface of the modified electrode with DIW was carried out. The structure of the modified electrode was confirmed by scanning electron microscopy (SEM; JEOL).

### 2.4. Electrochemical Measurement of the Modified Electrode

Electrochemical experiments on the fabricated SNP modified electrode covered by graphene oxide were performed using a potentiostat (CHI-660A, CH Instruments, Inc., Austin, TX, USA) to confirm the electrochemical signal enhancement. CV, DPV and the amperometric i-t method were performed using a three-electrode system composed of the SNP-modified electrode covered by graphene oxide as the working electrode, a platinum (Pt) wire electrode as the counter electrode and a silver/silver chloride (Ag/AgCl) electrode as the reference electrode. The electrochemical buffer solution (PBS solution) was used for the electrochemical measurement. The parameters of this experiment were a sensitivity of 1.0 × 10^−5^ A/V, scan rate of 100 mV/s, quiet time of 2 s and sample interval of 1 mV. The range of the applied potential voltage for CV and DPV was from −0.2 V to 0.6 V. Considering the amperometric i-t technique, −0.3 V, 0.1 s and 1.0 × 10^−5^ A/V were used for the respective initial potential, sampling interval and sensitivity. Chemical neurotransmitters, namely dopamine, uric acid and ascorbic acid, were dissolved in PBS at various concentrations of dopamine and 50 μM of both uric acid and ascorbic acid. 

## 3. Results and Discussion

### 3.1. Structure of the SNP Modified Electrode Covered by Graphene Oxide

The SNP modified electrodes covered by graphene oxide were fabricated using various concentrations of silver nitrate solution and graphene oxide solution with an electrochemical deposition technique in order to find the optimal size of SNPs and thickness of graphene oxide. A plastic chamber was used in both the fabrication process and dopamine detection. A constant inner surface area (19.6 mm length × 8.0 mm width) and a constant volume of silver nitrate, graphene oxide and dopamine could be achieved by using the plastic chamber. Since the volume of the solution was fixed, variables relating to the volume change were removed. The size of SNPs increased or decreased in proportion to the amount of time the voltage was applied for. Likewise, the density of the SNPs on the surface becomes more densely packed as the concentration of silver nitrate solution increased. However, at high a concentration of silver nitrate solution, the number of deposited SNP increased. This resulted in a non-uniform surface of the SNP modified electrode resulting in irregular electrochemical signals. For these reasons, the deposition of SNPs was optimized using a 50 mM silver nitrate solution and 5 s of voltage application time. In a similar way, various concentrations of graphene oxide solution and voltage application times were tested to optimize the condition of graphene oxide deposition. The SEM images of the surface of electrode became dimmer as the concentration of graphene oxide solution increased because of the increased amount of graphene oxide deposition. The voltage application time and sodium sulfate used as an electrolyte for graphene oxide deposition affected the thickness of the graphene oxide. Additionally, graphene oxide was deposited in a sheet structure and if the thickness was too large, the SNP surface would be covered to a great extent. This resulted in a decrease in the electrochemical signal of dopamine and therefore an optimized condition was required. As the concentration of the graphene oxide solution and the voltage application time increased, the thickness of the graphene oxide sheet increased similarly to the relationship between the size of the SNPs and the concentration of silver nitrate and voltage application time. From these results, the condition for optimal graphene oxide deposition was achieved using a 50 mg/mL graphene oxide solution, 30 s of voltage application time and a 0.1 M concentration of sodium sulfate. 

**Figure 2:**
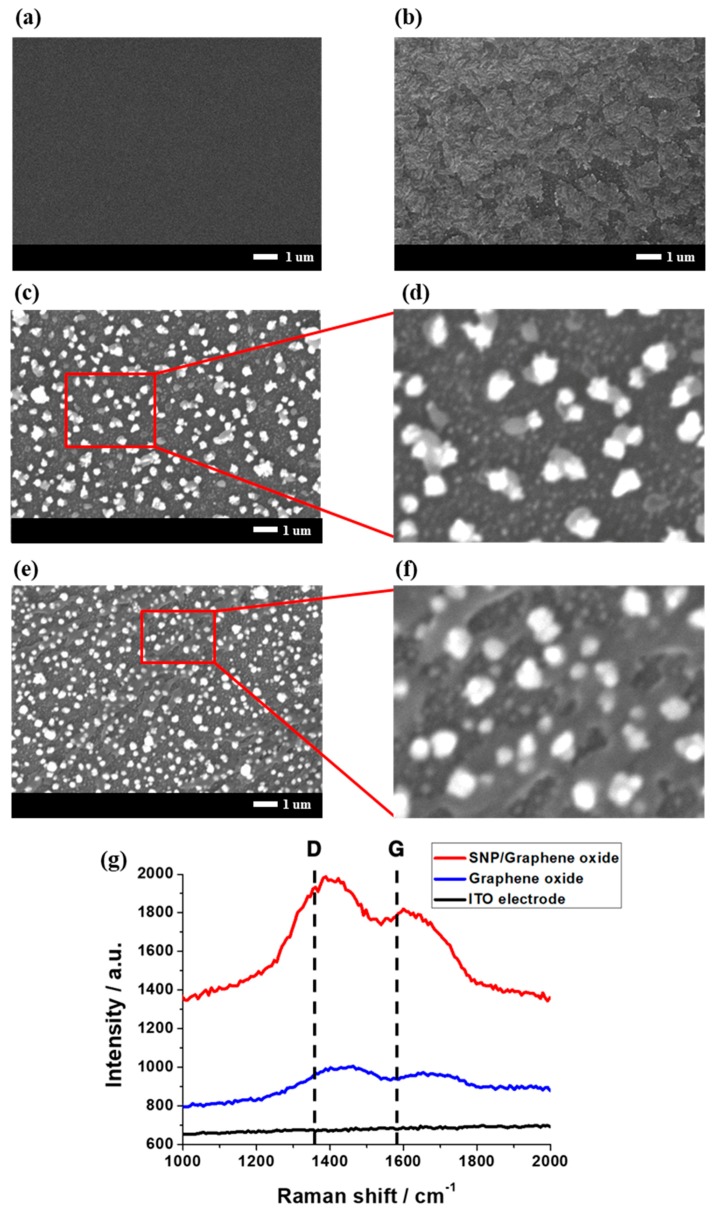
SEM images of the (**a**) ITO electrode, (**b**) ITO electrode covered by graphene oxide, (**c**) SNP modified electrode, (**d**) magnification of the SNP modified electrode, (**e**) SNP modified electrode covered by graphene oxide and (**f**) magnification of the SNP modified electrode covered by graphene oxide; (**g**) Raman spectroscopy of the ITO electrode, ITO electrode covered by graphene oxide and SNP modified electrode covered by graphene oxide.

**Figure 3:**
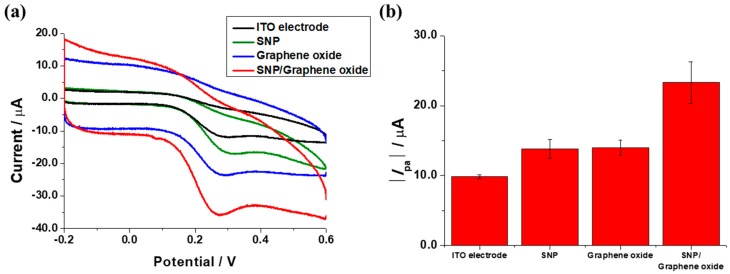
Electrochemical signal enhancement of the ITO electrode, SNP modified electrode, ITO electrode covered by graphene oxide and SNP modified electrode covered by graphene oxide with the addition of a 50 μM dopamine solution using (**a**) CV measurements and (**b**) comparison of the absolute I_pa_ values.

The morphology of the electrode is shown in Figure 2. The deposited SNPs and graphene oxide on the ITO electrode were confirmed by SEM. The SNPs were deposited spherically and had a diameter of about 100 to 200 nm, as shown in Figure 2c. On the other hand, graphene oxide was deposited as if the small pieces covered the electrode surface, as shown in Figure 2b. Lastly, the surface of the SNP modified electrode covered by graphene oxide was seen as dimmer than the SNP modified electrode without graphene oxide, as shown in Figure 2e. This result became clearer when the SEM image was magnified. As shown in Figure 2d,f, thin sheets of graphene oxide are observed above the spherical SNPs on the surface of the SNP modified electrode covered by graphene oxide, while the SNP modified electrode without graphene oxide contained no sheet above the SNPs. Furthermore, Raman spectroscopy was carried out to precisely confirm the existence of graphene oxide above the ITO electrode and SNPs. As shown in Figure 3g, D (1350 cm^−1^) and G (1580 cm^−1^) peaks were observed for the ITO covered by graphene oxide and SNP modified electrode covered by graphene oxide, while the bare ITO electrode possessed no peak. The G band was caused by vibration of the SP^2^-carbon system and the D band was caused by a structural deficit of the graphene oxide. From these results, the presence of the graphene oxide film on the SNP modified electrode was confirmed. Therefore, it could be confirmed that the surface of the ITO electrode, SNP modified electrode, ITO electrode covered by graphene oxide and SNP modified electrode covered by graphene oxide had been successfully fabricated.

### 3.2. Confirmation of Signal Enhancement of the SNP Modified Electrode Covered by Graphene Oxide

The enhancement of electrochemical signal was confirmed by comparison among the ITO electrode, SNP modified electrode, ITO electrode covered by graphene oxide and SNP modified electrode covered by graphene oxide. The concentration of dopamine was fixed at 50 μM for comparison. Dopamine was detected at the oxidation peak (0.276 V) by CV. The detection of dopamine was achieved by the adsorption and oxidation of dopamine to the electrode surface. This oxidation of dopamine is an irreversible electrochemical reaction. An oxide layer was thought to be formed when the electrolytic solution was cycled. Since oxide layers increase the adsorption of cationic species, the electrostatic force between the electrode surface and dopamine was increased by the formed oxide layer. Additionally, the achieved electrochemical signal could be unstable when the electrode surface was modified; however, the fabricated electrode surface was constant due to the use of the plastic chamber. For the quantitative assay, an anodic current peak (I_pa_) was used throughout the experiment. Figure 3a shows the CV measurements of the various electrodes at a 50 μM concentration of dopamine. The SNP modified electrode covered by graphene oxide provided the highest oxidation peak current, while lower oxidation peak currents were provided by the ITO electrode covered by graphene oxide, SNP modified electrode and ITO electrode, in decreasing order. Figure 3b shows the comparison of the absolute I_pa_ values provided by the various electrodes. The electrochemical property of electrode was enhanced compared to the ITO electrode, SNP modified electrode and ITO electrode covered by graphene oxide. This enhancement of the electrochemical peak current was induced by the properties of the SNP and graphene oxide [15]. Since SNP is well-known metal nanoparticle with high conductivity, such that they may provide amplification of electrochemical signals in order to clarify a current peak. Furthermore, graphene oxide was introduced for the enhancement of the electrochemical signal. Graphene oxide is a nearly nonconductive material; however, graphene oxide was reduced in the process of electrochemical deposition thus an oxide layer was made. Since the oxide layer increased the adsorption of dopamine, enhancement of the electrochemical dopamine signal was increased. Therefore, the properties of SNP and graphene oxide may provide an increased electrochemical signal detected by a modified electrode similarly to SNPs [16]. Finally, with the combination of the properties of SNPs and reduced graphene oxide, the electrochemical deposition of graphene oxide on a SNP surface would provide significantly enhanced electrochemical signals. Additionally, graphene oxide could protect the surface of the SNPs from surface oxidation [17,18,19].

### 3.3. Detecting the Performance of the SNP Modified Electrode Covered by Graphene Oxide

**Figure 4:**
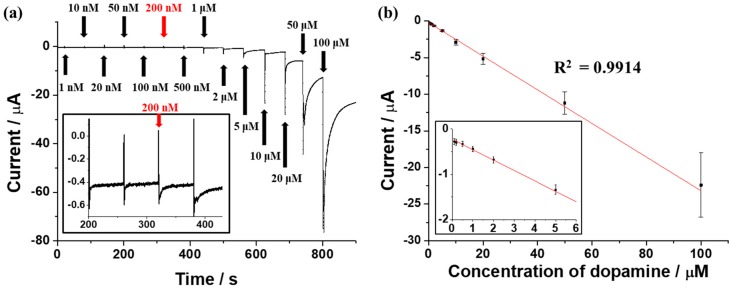
(**a**) Amperometric i-t dopamine response with the addition of various dopamine concentrations; (**b**) linear curve of the peak current values and different dopamine concentrations (*n* = 3).

The amperometric i-t technique was performed in order to determine the detecting performance of the SNP modified electrode covered by graphene oxide. To verify the detection limit of the SNP modified electrode covered by graphene oxide, the amperometric i-t technique was conducted with the addition of dopamine at various concentrations (0.001, 0.01, 0.02, 0.05, 0.1, 0.2, 0.5, 1, 2, 5, 10, 20, 50 and 100 μM). As shown in Figure 4a, the current value was maintained at about −0.28 μA until the addition of 0.1 μM of dopamine. However, as 0.2 μM of dopamine was added, the current value increased to −0.30 μA. After that, the current value increased as the concentration of the added dopamine was increased. With this result, the detection limit of the SNP modified electrode covered by graphene oxide was estimated to be 0.2 μM. The linearity of the peak current values and dopamine concentrations was confirmed by comparison of those two values obtained by the amperometric i-t technique. The peak current values of dopamine showed good linearity with the use of different dopamine concentrations in the range of 0.1 μM to 100 μM, as shown in Figure 4b. The coefficient of determination value, R^2^, was 0.9914, which indicates excellent linearity. Average values and error bars were obtained by the standard deviation (SD) of three measurements. The detection limit of this electrode was more sensitive and comparable to that of other electrodes used for dopamine detection, as shown in Table 1. Moreover, the linear range of the SNP modified electrode covered by graphene oxide was found to be superior to other electrodes such as the gold nanoparticle-graphene oxide modified electrode, as can be seen in Table 1.

### 3.4. Electrochemical Selective Detection of Dopamine in the Presence of Uric Acid and Ascorbic Acid

**Figure 5:**
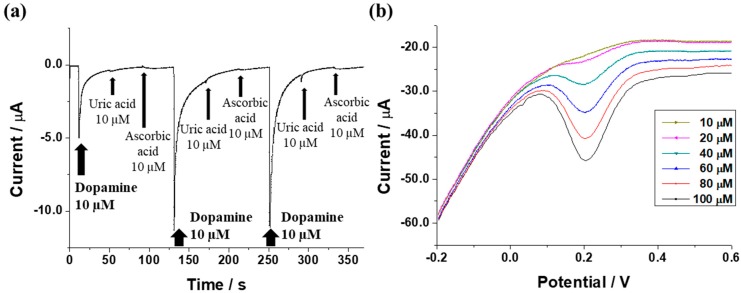
(**a**) Amperometric i-t measurement with the continuous addition of 10 μM of each of dopamine, uric acid and ascorbic acid; (**b**) DPV for the concentration of dopamine ranging from 10 μM to 100 μM in the presence of 50 μM of both uric acid and ascorbic acid.

Uric acid and ascorbic acid coexist with dopamine in the body and demonstrate similar electrochemical properties to those of dopamine [22,23]. Thus, the selective electrochemical detection of dopamine in the presence of uric acid and ascorbic is an important factor when considering the practical use of an SNP modified electrode covered by graphene oxide. Figure 5a shows the efficient selective amperometric response of the SNP modified electrode covered by graphene oxide with additions of 10 μM of each of dopamine, uric acid and ascorbic acid, continuously. The SNP modified electrode covered by graphene oxide demonstrated an amperometric response to dopamine and did not respond to uric acid and ascorbic acid. Figure 5b showed successful DPV measurements of dopamine obtained from the SNP modified electrode covered by graphene oxide in the presence of uric acid and ascorbic acid. The concentration of dopamine was varied from 10 μM to 100 μM and concentrations of uric acid and ascorbic were fixed to 50 μM. The electrochemical peak current was increased without disturbance of the uric acid and ascorbic acid as the concentration of dopamine increased. From these results, the electrochemical selective detecting performance of the SNP modified electrode covered by graphene oxide was confirmed.

## 4. Conclusions

In this study, an electrochemical biosensor based on electrochemically deposited SNP and graphene oxide was fabricated for use in dopamine detection with an enhanced electrochemical signal, high selectivity and high sensitivity. To achieve the electrochemical signal enhancement, for the first time, SNP was electrochemically deposited onto the ITO electrode followed by the electrochemical deposition of graphene oxide. Compared with previous methods that simply mix SNPs and graphene oxide and deposit the SNPs on graphene oxide, this newly proposed method was attempted. The surfaces of modified electrodes were confirmed by SEM. The electrochemical signal enhancement of the modified electrodes was confirmed by CV. With the deposition of graphene oxide on the SNP surface, the SNP modified electrode covered by graphene oxide showed a much higher enhancement of the electrochemical signal than the SNP- or graphene oxide-modified electrodes. The high selectivity of this biosensor was also confirmed by DPV and an amperometric i-t technique. Dopamine in concentrations ranging from 10 μM to 100 μM was successfully detected in the presence of 50 μM of uric acid and 50 μM of ascorbic acid. The amperometric i-t dopamine detection showed high selectivity towards dopamine. Lastly, the high sensitivity of this electrode was confirmed by the amperometric i-t technique from dopamine concentrations of 0.1 μM to 100 μM as expressing linearity and 0.2 μM as the detection limit. In conclusion, our newly developed biosensor could provide a method for the fabrication of a sensing platform with an enhanced electrochemical signal, high selectivity and high sensitivity in order to detect dopamine in various fields.

## Figures and Tables

**Table 1:** Comparison of some electrochemical characteristics of different graphene-based or SNP-based electrodes for the detection of dopamine.

Electrode	Methods	Linear Range (μM)	Detection Limit (μM)	Reference
pGO ^1^-GNP ^2^-pGO	CV, AM ^9^	0.1–30	1.28	[13]
Pdop ^3^@Gr ^4^/MWCNTs ^5^	DPV	7–297	1.0	[20]
Ag-CNT ^6^/CPE ^7^	DPV	0.8–64	0.3	[21]
SNP/GO ^8^	CV, AM	0.1–100	0.2	This work

^1^ Porous graphene oxide; ^2^ Gold nanoparticle; ^3^ Polydopamine; ^4^ Graphene; ^5^ Multi-walled carbon nanotubes; ^6^ Carbon nanotube; ^7^ Carbon-paste electrode; ^8^ Graphene oxide; ^9^ Amperometry.

## Supplementary Materials

The following are available online at www.mdpi.com/1424-8220/17/12/2771/s1, Figure S1: FE-SEM images and EDS of (a) SNP modified electrode and (b) SNP modified electrode covered by graphene oxide, Figure S2: Comparison of CV peaks of dopamine with uric acid and uric acid, Figure S3: (a) Linear sweep voltammetry and (b) comparison of peak current of different electrodes for electroactive area comparison, Figure S4: Amperometric i-t curve of SNP modified electrode covered by graphene oxide with 4% human serum, Figure S5: Electrochemical peak current comparison between SNP modified electrode and SNP modified electrode covered by graphene oxide at 0 h and 24 h, Table S1: Parameters comparison between different electrodes.

## Abbreviations

PD	Parkinson’s Disease
ADHD	Attention Deficit Hyperactivity Disorder
HPLC	High Performance Liquid Chromatography
SNP	Silver Nanoparticle
ITO	Indium Tin Oxide
CV	Cyclic Voltammetry
DPV	Differential Pulse Voltammetry
PDMS	Polydimethylsiloxane
DIW	Deionized Water
SEM	Scanning Electron Microscopy
SD	Standard Deviation
