# Supplementary material for: Silver Nanoparticle Modified Electrode Covered by Graphene Oxide for the Enhanced Electrochemical Detection of Dopamine
Source: Sensors (Basel). 2017 Nov 29;17(12):2771. doi: 10.3390/s17122771 (PMC5750767; doi:10.3390/s17122771)
Supplement: Supplementary file 1 [file sensors-17-02771-s001.pdf]

SUPPLEMENTARY ONLINE MATERIAL To Sensors

Title: Silver Nanoparticle Modified Electrode Covered by Graphene Oxide for the Enhanced Electrochemical Detection of Dopamine

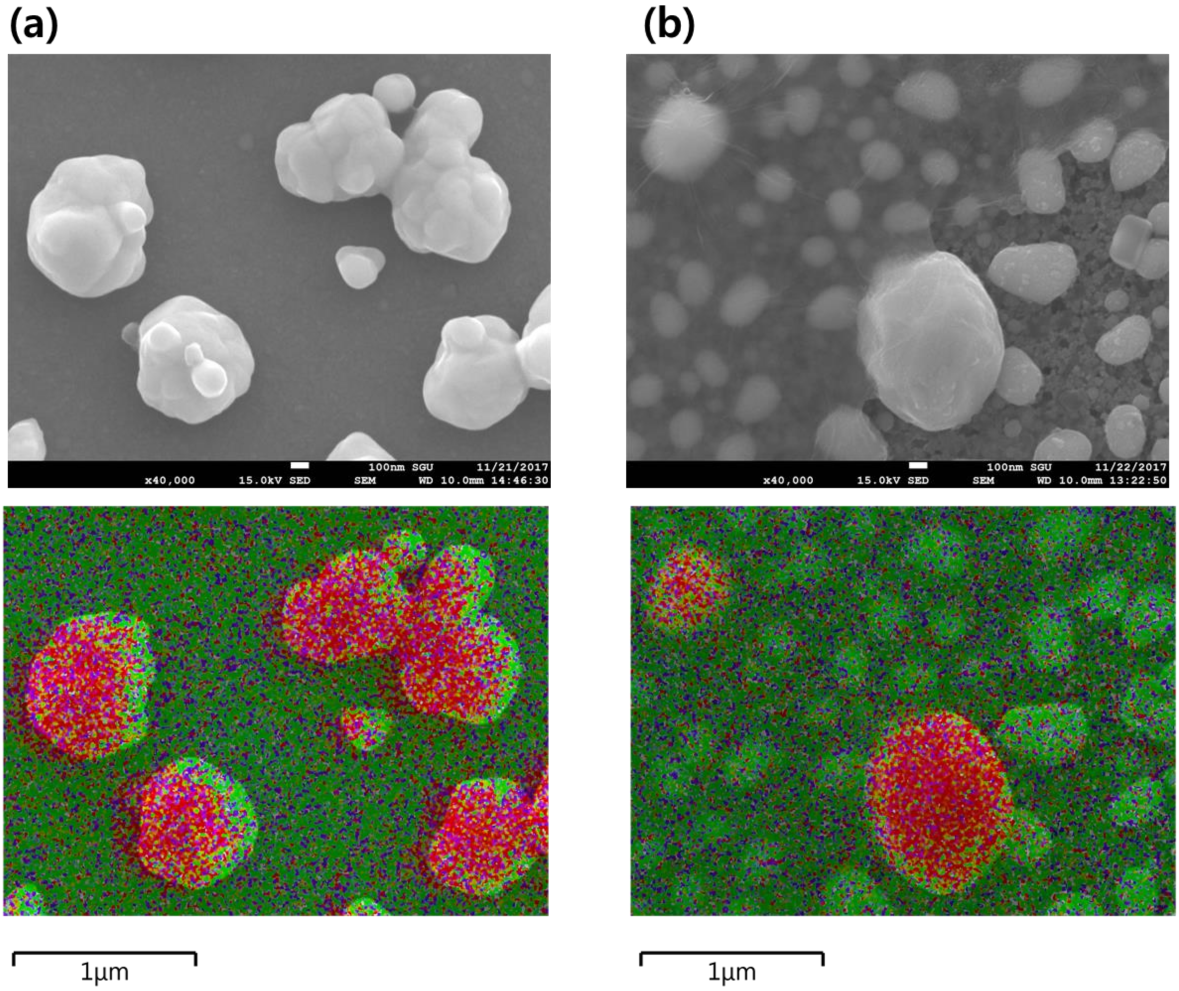

Figure S1. FE-SEM images and EDS of (a) SNP modified electrode and (b) SNP modified electrode covered by graphene oxide. (green: In, red: Ag, blue: C)

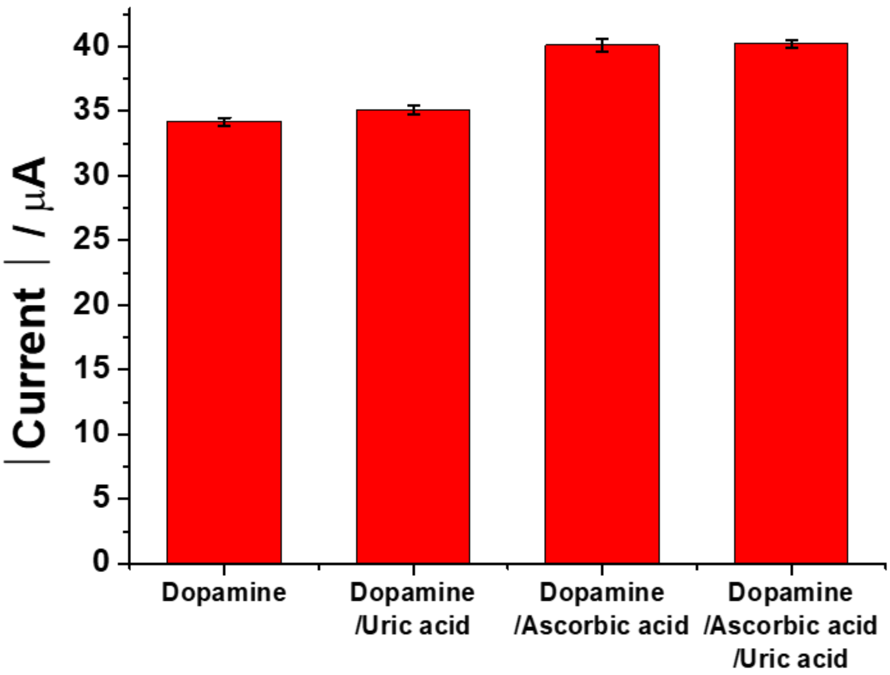

Figure S2. Comparison of CV peaks of dopamine with uric acid and uric acid.

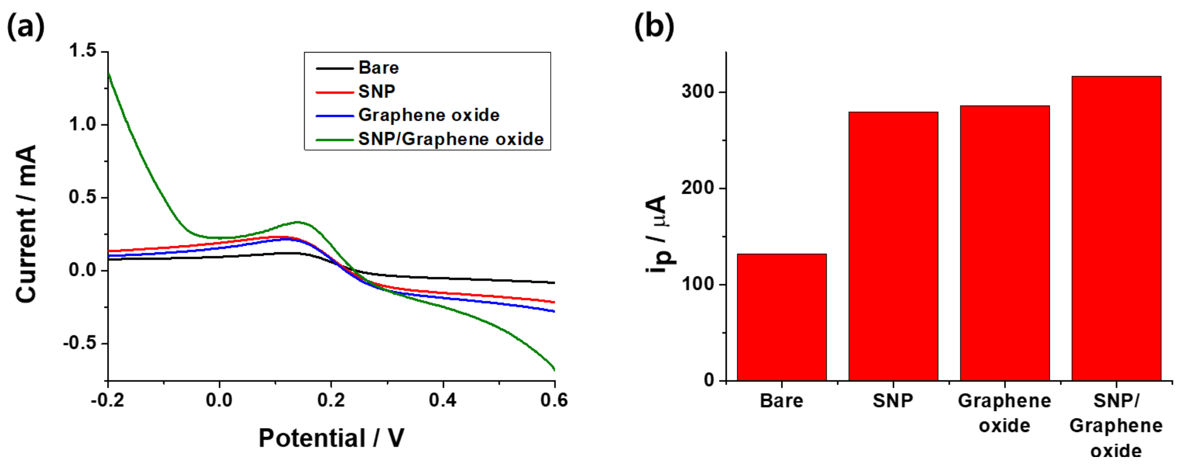

Figure S3. (a) Linear sweep voltammetry and (b) comparison of peak current of different electrodes for electroactive area comparison.

Randles-Sevcik equation:

$$i_p = 268{,}600 n^{\frac{3}{2}} A D^{\frac{1}{2}} C v^{\frac{1}{2}}$$

i_p: peak current (A)

n: the number of electrons transferred (n=1)

A: electrode surface area (cm^2)

D: diffusion coefficient (0.721 cm^2 s^{-1})

C: reactant concentration (mol cm^{-3})

v: scan rate (V s^{-1})

D was calculated as 0.721 cm^2 s^{-1}. Also, n was 1, C was 1 mmol/L, and v was 0.1 V/s. i_p for each electrode was 1.319×10^{-4} A for ITO electrode, 2.793×10^{-4} A for SNP modified electrode, 2.862×10^{-4} A for ITO electrode covered by graphene oxide and 3.166×10^{-4} A for SNP modified electrode covered by graphene oxide.

With these results, the Randles-Sevcik equation becomes simple as

$$i_p \propto A.$$

With this equation, the real electroactive area A is proportional to i_p. The geometrical surface area was constant since the plastic chamber was used, so, we determine that increase of electroactive area had responsible for signal enhancement.

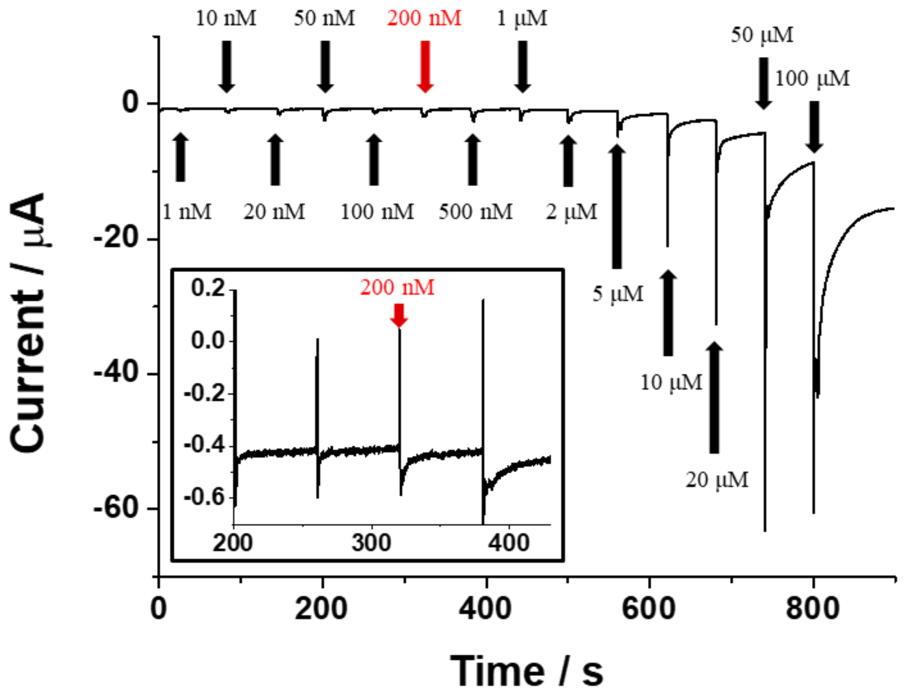

Figure S4. Amperometric i-t curve of SNP modified electrode covered by graphene oxide with 4 % human serum.

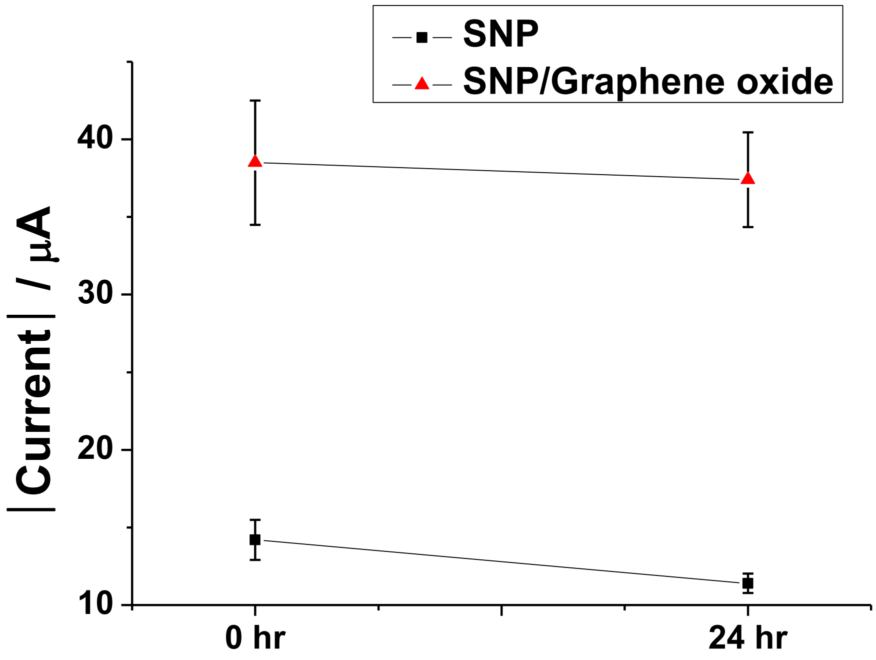

Figure S5. Electrochemical peak current comparison between SNP modified electrode and SNP modified electrode covered by graphene oxide at 0 hr and 24 hr. The concentration of dopamine solution was 50 μM. The electrochemical signal of SNP modified electrode covered by graphene oxide dropped 2.9 %, while that of SNP modified electrode dropped 19.2 %.

Electrode	Methods	R^2	RSD%	Reference
GNCs/CMG/GCE	CV, AM	0.993	2.7 %	[1]
Co^{II}-CN-Cu^{II}	DPV, AM	0.9966	1.9 %	[2]
PPy/CNTs-MIP	CV, DPV	0.9998	2.13 %	[3]
SNP/GO	CV, AM	0.9914	1.59 %	This work

Table S1. Parameters comparison between different electrodes.

1. Daemi, S.; Ashkarran, A.A.; Bahari, A.; Ghasemi, S. Gold Nanocages Decorated biocompatible Amine Functionalized Graphene as an Efficient Dopamine Sensor Platform. *J. Colloid Interface Sci*. **2017**, *494*, 290-299, http://dx.doi.org/10.1016/j.jcis.2017.01.093.
2. Karikalan, N.; Velmurugan, M.; Chen, S.M.; Chelladurai, K. A Copper Hexacyanocobaltate Nanocubes based Dopamine Sensor in the Presence of Ascorbic Acid. *RSC Adv*. **2016**, *6*, 48523, DOI: 10.1039/c6ra05810h.
3. Qian, T.; Yu, C.; Zhou, X.; Ma, P.; Wu, S.; Xu, L.; Shen, J. Ultrasensitive Dopamine Sensor based on Novel Molecularly Imprinted Polypyrrole Coated Carbon Nanotubes. *Biosens. Bioelectron*. **2014**, *58*, 237-241, http://dx.doi.org/10.1016/j.bios.2014.02.081.
